# *In Vitro* Cytotoxic Activity against Breast, Cervical, and Ovarian Cancer Cells and Flavonoid Content of Plant Ingredients Used in a Selected Thai Traditional Cancer Remedy: Correlation and Hierarchical Cluster Analysis

**DOI:** 10.1155/2020/8884529

**Published:** 2020-11-17

**Authors:** Thammarat Tuy-on, Arunporn Itharat, Ponlawat Maki, Pakakrong Thongdeeying, Weerachai Pipatrattanaseree, Buncha Ooraikul

**Affiliations:** ^1^Graduate School, Faculty of Medicine, Thammasat University, Bangkok, Pathumthani 12120, Thailand; ^2^Department of Applied Thai Traditional Medicine, Faculty of Medicine, Thammasat University, Bangkok, Klongluang, Pathumthani 12120, Thailand; ^3^Center of Excellence in Applied Thai Traditional Medicine, Faculty of Medicine, Thammasat University, Klongluang, Bangkok, Pathumthani 12120, Thailand; ^4^Regional Medical Science Center 12 Songkhla, Department of Medical Sciences, Ministry of Public Health, Muang, Songkhla 90100, Thailand; ^5^Department of Agricultural Food and Nutritional Science, Faculty of Agricultural Life and Environmental Sciences, University of Alberta, Edmonton, AB, Canada

## Abstract

This study aimed to investigate *in vitro* cytotoxic activity of selected plant ingredients from a traditional Thai remedy for the treatment of cancer patients against cancer cells occurring in women such as MCF-7 (breast cancer), SKOV3 (ovarian cancer), and HeLa (cervical cancer) cell lines. The plants and the remedy were macerated with 95% ethanol and boiled in water. Cytotoxic activity of the extracts was analyzed by SRB assay. Total flavonoid contents of the extracts were determined and their correlation with cytotoxic activity was evaluated. The hierarchical cluster analysis (HCA) was used to classify the extracts by their cytotoxic characteristics. A total of 66.7% of the plants was active against the tested cancer cell lines. Among the 44 plants in the remedy used for cancer treatment, nine plants that are also used in Thai cuisine exerted significant cytotoxicity against tested cancer cell lines. Eleven plants in the remedy were active against at least one of the tested cancer cell lines. All extracts were grouped into three groups and illustrated as heat map and hierarchical dendrogram. Total flavonoid content showed weak or no correlation with cytotoxic activity. *A. dahurica, F. albopurpurea,* and *T. indica* selectively exerted potent cytotoxic activity against MCF-7 with SI value more than 6. *A. galanga, P. amarus*, *L. striatum*, *H. indicum,* and *F. vulgare* exerted moderate cytotoxicity to all tested cell with low toxicity to normal cells. The correlation and HCA performed in this study provided an alternative way to investigate biological activities of plant ingredients in polyherbal traditional remedies.

## 1. Introduction

Cancers of breast, cervical, and ovarian are common cancers occurring in the female population. Breast cancer is the most common and the most leading cause of cancer death among women worldwide. Cervical and ovary cancers are the fourth and seventh most common, respectively [1]. Although each cancer has its own established treatment guidelines, the conventional treatments are surgery, radiation, and chemotherapy. The current treatments, particularly anticancer drugs, may be inadequate for patients due to some severe side effects arising from toxicity on normal cells or tissues. These side effects of chemotherapy often affect patient's quality of life [2]. Therefore, novel substances or plant extracts need to be developed for the treatment and prevention of cancers.

Herbal medicines for cancer treatment are known to cancer patients. These medicinal plants have become increasingly recognized either as alternative medicines for cancer treatment or dietary supplements for cancer prevention. In Thai traditional medicine (TTM) prescribed by traditional doctors, several medicinal plants are combined as remedies [3, 4]. TTM is a cultural heritage and indigenous wisdom that has been used by Thai people for over a thousand years. It utilizes several herbs rather than a single herb to design a remedy for cancer treatment. The selected TTM remedy in this study was a cancer remedy prescribed at the Jitmeatta Mercy Foundation for Cancer Patients of Thailand (JFCT), Phetchaburi province, Thailand. This remedy is composed of 44 plants mixed by equal weight. This traditional cancer remedy was prepared by the decoction method and it was used to treat cancer patients more than 30 years ago. Previous research showed that almost all cancer patients treated at JFCT were women, so this research pointed to prove that this cancer remedy can cure cancers in women [5].

Phytochemical compounds in herbal medicines play an important role in the expression of biological and pharmacological activities. Flavonoids, a group of phenolic compounds, provide potential benefits in health promotion and prevention of several chronic diseases including cancer. Due to the variation of substitutions on the skeletal structure of flavonoids, an extremely diverse range of flavonoid derivatives in herbal medicines have been discovered [6]. Many flavonoids showed cytotoxic activity and antitumor, such as epigallocatechin gallate and quercetin [7].

Thus, the aim of this study was to investigate *in vitro* cytotoxic activity of the extracts of this TTM remedy and its 44 plant ingredients in this remedy against commonly occurring cancers in women, i.e., MCF-7 (breast cancer), SKOV3 (ovarian cancer), and HeLa (cervical cancer). The total flavonoid content of the extracts was also studied, and its correlation with cytotoxic activity was also evaluated. The statistical cluster analysis was used to visualize the cytotoxic pattern of plants and remedies. This study also evaluated an alternative method for screening the cytotoxic activity of plant ingredients in polyherbal remedies used in traditional medicines. The correlation of cytotoxic activity with total flavonoid content may be used in the search for cytotoxic compounds from flavonoids against cancers occurring in women in the future.

## 2. Materials and Methods

### 2.1. Plant Materials

All plants were collected from Phetchaburi Province, Thailand, in 2018. The herbarium specimens were prepared and deposited at the herbarium of Southern Center of Thai Medicinal Plants at the Faculty of Pharmaceutical Science, Prince of Songkla University, Songkhla, Thailand, and the relevant voucher numbers are shown in Table 1.

### 2.2. Preparation of Plant and TTM Extracts

Each plant was washed, thinly sliced, dried in an oven at 45°C, and powdered. Dried plants (1000 g) were macerated with 3.0 L of 95% ethanol for 3 days, filtered, and evaporated to dryness. The selected traditional Thai cancer remedy was prepared according to the proportion used at the JFCT foundation by combining an equal weight of each dried plant, mixed together, and powdered. The resulting remedy was extracted by two methods, maceration (as described above), and decoction. The decoction method was carried out by boiling the remedy (1000 g) with an equal amount of water for 15 minutes and the aqueous extract filtered and evaporated to dryness with a freeze drier. The ethanolic extract of TTM was named TTE and the aqueous extract obtained from decoction was named TTW. The %yield was calculated (Table 1) and all extracts were kept at −20°C until use.

### 2.3. Cell Lines and Culture

Breast cancer cell lines (MCF-7 ATCC NO HTB-22), ovarian cancer (SKOV-3 ATCC NO HTB-77), and cervical cancer (HeLa ATCC NO CCL-2) were purchased from the American Type Culture Collection (Manassas, VA, USA). Normal human keratinocyte immortal cell line (HaCat) was purchased from the CLS cell line service (No. 300493-SF).

MCF-7 and HeLa cell lines were cultured in minimum essential medium (MEM). SKOV-3 was cultured in Roswell Park Memorial Institute medium 1640 (RPMI-1640) and HaCat was cultured in Dulbecco's Modified Eagle's Medium (DMEM). All cultured media were supplemented with 10% fetal bovine serum and 1% penicillin-streptomycin. The cells were incubated in 5% CO_2_ at 37°C and 95% humidity.

### 2.4. In Vitro Assay for Cytotoxic Activity

Cytotoxicity of the extracts was investigated by using sulphorhodamine B (SRB) assay according to the method previously described [8, 9]. Briefly, cells were seeded in a 96-well microtiter plate and incubated for 24 hours. Each extract was diluted by DMSO to produce stock solution at a concentration of 10 mg/ml except TTW, which was diluted by water. The stock solution was diluted serially by culture medium to produce a working solution (2, 20, 100, and 200 *μ*g/ml). The seeded cells were treated with various concentrations of working solution of plant and remedy extracts and incubated for 72 hours. The final concentrations of extract in the seeded cell were 1, 10, 50, and 100 *μ*g/ml and the maximum final concentration of DMSO in the seeded cell was 1%v/v. The medium was replaced with cell culture media and the plates were continuously incubated at 37°C for three days in order to observe cytotoxicity after a recovery period. The cells were fixed with 40% trichloroacetic acid and the plates left at 4°C for 45 min. The media were removed and the cells were stained with 0.4% SRB in acetic acid. The %survival was determined by colorimetric measurement at 492 nm and calculated by the following equation. The half inhibitory concentrations (IC_50_) of the extracts were calculated by using GraphPad Prism software (CA, USA)

(1) $$\begin{array}{c} \text{Inhibition}\left(\text{\%}\right)=\left(\frac{\text{ODcontrol}-\text{ODsample}}{\text{ODcontrol}}\right)\times 100. \end{array}$$

### 2.5. Total Flavonoid Content

Total flavonoid content was measured by the aluminum chloride colorimetric assay [10]. Briefly, an aliquot (1 mL) of the extracts or standard solution of quercetin (20, 40, 60, 80, 100, 120, and 240 mg/L) was added to a volumetric flask. To the flask were added 75 *μ*L of 5% NaNO_2_ and 150 *μ*L of 10% AlCl_3_. After 5 min, 500 *μ*L of 1 M NaOH was added, and the volume was adjusted by H_2_O. The solution was mixed well, and the absorbance was measured against the prepared reagent blank at 510 nm. The concentration of total flavonoid content in the test samples was calculated from the calibration plot (*Y* = 0.0162*x* + 0.0044, *r*^2^ > 0.999) and expressed as mg quercetin equivalent/g of dried plant. All the determinations were carried out in triplicate.

### 2.6. Statistical Analysis

All experiments were carried out by three independent experiments, and the data were reported as the mean and standard error of means. The correlation of total flavonoid content and cytotoxic activity was plotted and the correlation coefficient (*r*) calculated by using Microsoft® excel. The similarity of cytotoxic activity to the three cancer cells of the tested plants was classified using the hierarchical cluster analysis (HCA) and visualized as a heat map using Heatmap Illustrator (HemI) version 1.0 software [11].

## 3. Results

### 3.1. Cytotoxic Activity

The cytotoxic activity of the plant and remedy extracts is shown in Tables 2 and 3. The extracts obtained from the selected cancer remedy, TTW and TTE, were not active (IC_50_ > 100 *μ*g/ml). However, TTE exerted moderate activity against MCF-7 with IC_50_ of 52.33 ± 2.05 *μ*g/ml. Of the 46 extracts tested, 30 (65.2%) exerted cytotoxic activity against the cancer cells with various degrees of potency. Four plant extracts exerted potent cytotoxic activity against MCF-7 with IC_50_ values less than 20 *μ*g/mL, including *A. dahurica, M. siamensis, F. albopurpurea,* and *T. indica*. The other 26 extracts exerted low to moderate cytotoxic activity. With regard to normal cells (HaCat), 15 extracts showed no cytotoxic activity with IC_50_ values more than 100 *μ*g/mL. The selective index (SI) of the extracts was calculated. SI is the ratio between IC_50_ values of HaCaT (normal cell) and cancer cell lines. The greater the SI value, the more selective it is. SI value more than 2 indicates that the herbal extract is toxic against cancer cells but less toxic to normal cells. SI value less than 2 indicates the general toxicity of the extract [12]. Based on this criterion, the extracts of*. A. dahurica*, *F. albopurpurea, M. siamensis*, *C. halicacalum, C. papaya*, *K. galanga*, *P. cablin, and T. indica* were remarkably selective to breast cancer (MCF-7) cells. Plant extracts which exerted activities against all tested cancer cells were from *A. galaga, L. striatum, P. amarus,* and *H. indicum*.

### 3.2. Total Flavonoid Content and Correlation with Cytotoxic Activity

**Figure 1:**
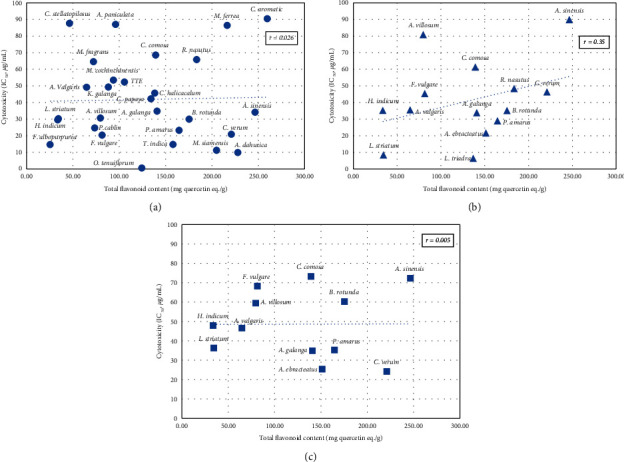
Scatter plots of flavonoid content and cytotoxicity of the plant extracts (IC_50_ < 100 *μ*g/ml) against cancer cell lines. *r* = correlation coefficient, (a) = MCF-7, (b) = SKOV, (c) = Hela.

**Figure 2:**
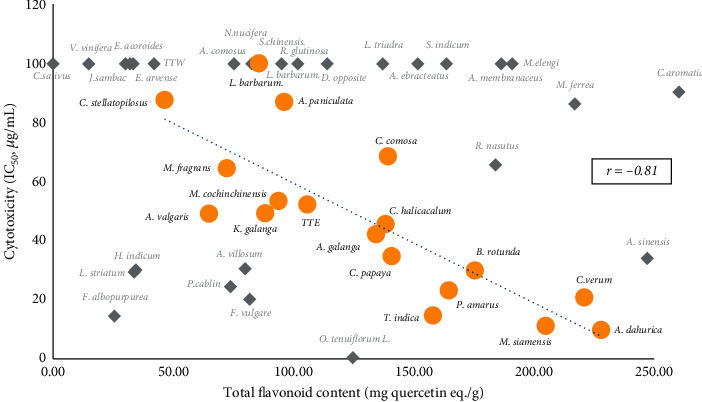
Scatter plot of cytotoxicity to MCF-7 and total flavonoid content of some selected plant extracts. *r* = correlation coefficient. (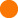) = the selected plant extracts that showed the correlation between total flavonoid content and cytotoxicity with correlation coefficient (*r*) value −0.81; (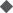) = the plant extracts that showed lesser correlation.

The total flavonoid content of the extracts is shown in Table 3. *C. aromatica* showed the highest content (259.7 ± 3.21 mg quercetin eq./g) followed by *A. sinensis* (259.70 ± 2.59 mg quercetin eq./g) and *A. dahurica* (227.95 ± 5.69 mg quercetin eq./g). The ethanolic extract of the Thai traditional remedy showed total flavonoid content of 105.67 ± 4.33 mg quercetin eq./g. The correlation between flavonoid content and cytotoxic activity of the extracts was investigated. Total flavonoid contents of the extracts were plotted against their cytotoxic activities, and the correlation coefficients (*r*) were evaluated. The results showed that there was no correlation between the total flavonoid content of the extracts and their cytotoxic activity against all tested cell lines (*r* < 0.35) (Figure 1). However, in MCF-7, when some outlier data were excluded, there was a strong correlation between flavonoid content and cytotoxicity with *r* value −0.81 (Figure 2).

### 3.3. Hierarchical Cluster Analysis

**Figure 3:**
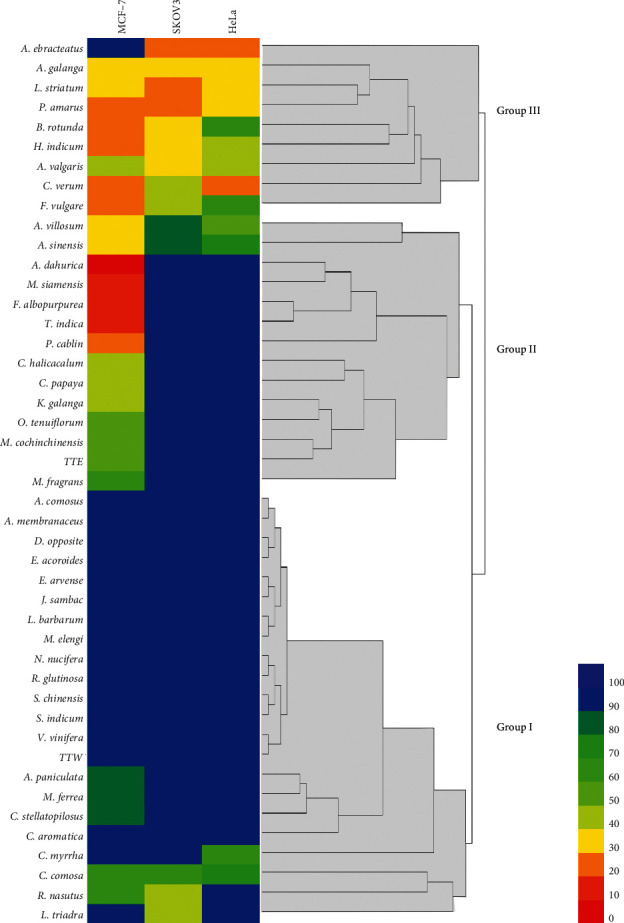
Heat map and hierarchical cluster analysis of plant and remedy extracts. Color scale bar showed a range of IC_50_, red color bar represents more potent cytotoxic activity (0–10 *μ*g/ml) incrementing to a blue color bar which represents weak cytotoxic activity (90–100 *μ*g/ml).

HCA is a multivariate statistical method to evaluate the pattern of data, which is classified into clusters according to their similar characteristics. The similarity or dissimilarity of the extracts is illustrated, as shown in the dendrogram (Figure 3). In this study, 44 plant extracts and two Thai traditional remedy extracts were classified by HemI software based on their cytotoxic activity against all tested cancer cells. The euclidean metric was selected to evaluate the similarity of the cytotoxic pattern. The heat map was generated to visualize the intensity of cytotoxic activity, and the linkages of similar extracts were shown in the horizontal dimension. As shown in Figure 3, HCA classified 46 extracts into three main groups. The extracts expressing low cytotoxicity against cancer cells were clustered into group I. Group II was composed of the extracts that selectively exhibited cytotoxicity against MCF-7. The extracts showing cytotoxicity against two or more cancer cells were clustered in group III.

## 4. Discussion

There has been an increasing interest in herbal medicine as an alternative treatment and prevention of cancer. Traditional Thai medicine is an alternative healthcare approach utilizing medicinal plants as a component in the holistic treatment of cancer patients [3]. In this study, we investigated the cytotoxic activity of selected Thai medicinal plants and remedy against cancer cells commonly occurred in women: breast (MCF-7), ovarian (SKOV3), and cervical cancer (HeLa). In the cytotoxic screening of 44 plants and two TTM extracts, the results were analyzed by HCA and visualized them into a heat map (Figure 3). Three major groups of the extracts were categorized by their cytotoxicity characteristics. Group I is composed of plant extracts which exerted less or no cytotoxic activity. Group II was the extracts that selectively exerted cytotoxicity against MCF-7. The extracts with moderate to high activity against all cancer cells were clustered into group III. With regard to the extracts obtained from the selected cancer remedy, TTW was not active against all tested cells, while TTE exerted moderate cytotoxicity against MCF-7. These results indicated that the cytotoxic compounds were extracted from the remedy in a small amount. However, the results of cancer treatment by TTM may occur by the accumulation of the cytotoxic compounds in the body when it was taken for long-term multiple doses. In TTM, many plants in remedy are used as many functions or biological activities related to cancer such as antiinflammation, increase immunology, antimicrobial, antioxidant activities. Another reason, TTM extract may help with another mechanism on cancer treatment such as increased immunology or angiogenesis, when this remedy was absorbed in the patient's body. Thus, this suggestion should be clarify further studied including *in vivo* and clinical trials investigation.

According to the heat map and HCA shown in Figure 3 along with IC_50_ and SI values presented in Tables 1 and 3, *A. dahurica, F. albopurpurea,* and *T. indica* exerted potent activity against MCF-7 with IC_50_ less than 20 *μ*g/ml and SI more than 6. *M. siamensis* exerted potent cytotoxicity against MCF-7 with SI more than 2. The results indicated that these extracts might have particularly beneficial effects for use in breast cancer treatment and prevention. *A. galanga, P. amarus,* and *L. striatum* exerted moderate cytotoxicity (IC_50_ > 20 *μ*g/ml) against all tested cancer cells with low toxicity to normal cells (SI > 2). *H. indicum* and *F. vugare* also selectively exerted mild to moderate cytotoxicity against the cancer cells.

Flavonoids, a group of phenolic compounds found in several medicinal plants, have a diverse range of derivatives depending on the substituted groups on their skeletal structure. This variety of flavonoids make the medicinal plants more valuable for their role in health promotion and prevention of several chronic diseases including cancer. In this study, we investigated the total flavonoid contents of the plants and analyzed their correlation with cytotoxicity against cancer cells. As shown in Figure 1, the total flavonoid content of the extracts exhibited weak or no correlation to cytotoxicity against all tested cancer cells with coefficient (*r*) less than 0.35. However, in MCF-7, when the outlier data were excluded and the correlation recalculated, some extracts showed a strong correlation between total flavonoid content and cytotoxicity with the coefficient of −0.81 (Figure 2). This indicated that flavonoids found in plants such as *T. indica*, *B. rotunda, and A. dahurica* might be related to their cytotoxicity against MCF-7.

Several flavonoids from *T. indica* have been reported, including vitexin, isovitrexin, orientin, and iso-orientin [13]. A previous report showed that vitexin and orientin exhibited mild to moderate cytotoxic activity against MCF-7 cell line with IC_50_ more than 50 *μ*g/ml [14]. Another report showed that the rhizome of *B. rotunda* contained flavonoids and chalcone compounds such as alpinetin, pinocembrin, cardamonin, pinostrobin, and panduratin A [15]. A cyclohexenyl chalcone, panduratin A, in *B. rotunda* showed potent cytotoxic activity against MCF-7 with IC_50_ of 3.75 *μ*g/ml [16]. *A. dahurica* showed high flavonoid content and exerted potent cytotoxic activity against MCF-7. The major constituents of *A. dahurica* were furocoumarins, imperatorin, phelloptorin, and isoimperatorin [17]. Imperatorin and isoimperatorin exhibited low to moderate cytotoxic activity against L1210, HL-60, K562, and B16F10 cell lines [18]. Components found in *M. siamensis* are coumarins such as mammea A, mammea B, mammea E, and other derivatives. Mammea A/AA exerted potent cytotoxic activity against breast cancer (MDA-MB-231) [19]. In *P. amarus,* the major chemical components are lignans. Crude methanolic extract of *P. amarus* exerted cytotoxic activity against MCF-7 and could reduce invasion, migration, and adhesion [20].

*C. aromatica* showed the highest content of flavonoids; however, it exerted mild cytotoxicity to all tested cancer cells indicating that the flavonoids in *C*. *aromatica* were not related to cytotoxicity against the cancer cells. Major chemical constituents found in *C*. *aromatica* were volatile terpenes, sesquiterpenes, and curcuminoids. These compounds may react with the chemicals used in the total flavonoid assay leading to the overestimation of the values [21]. *C. verum* also showed high content of flavonoids, the major components being cinnamaldehyde and methyl eugenol [22].

Among the plants that showed high total flavonoid content, *C. aromatica* extract which possessed the highest flavonoid content exerted mild cytotoxic activity while *A. dahurica* exerted potent cytotoxicity. This indicated that the flavonoids in *C. aromatica* differed from those in *A. dahurica,* leading to the differences in their cytotoxic activity. In phytochemical reviews, the major constituent in *C. aromatica* is terpenes, while in *A. dahurica* are furocoumarins. These indicated that these compounds might cause overestimation of total flavonoid content, which underscores the limitation of this study. Hence, the determination of the total flavonoid content of the plants alone may be insufficient to describe the correlation with their biological activities. Therefore, more in-depth phytochemical studies should be pursued by more powerful analytical methods and instruments.

## 5. Conclusion

Among the 44 plants in a selected cancer remedy used for cancer treatments, 17 plants are food ingredients in Thai cuisine and nine of them exerted significant cytotoxicity against the tested cancer cell lines. Eleven plants normally used in TTM were active against at least one of the cancer cell lines, while 66.7% of all extracts were active against all the tested cell lines. The remaining plant extracts may not have any cytotoxic activity but may be necessary adjuvants according to the TTM theory which considers the correction of the imbalance of body functions as an important aspect in designing a medicine. The correlation and HCA studies provided an alternative way to investigate the biological activities of plant ingredients in polyherbal traditional remedies. This method was designed to predict the correlation between their active components and biological activities. However, the results may not show a strong correlation between the active components in traditional remedies or mixed herbs and their cytotoxicity against cancer cells. This is because only some herbs in the remedy possess cytotoxic effects while the others are included to balance other physiological functions in the body to improve patient's well-being. Therefore, *in vivo* studies are necessary to elucidate the true efficacy of herbal medicines. Hierarchical cluster analysis can help interpret the results of *in vitro* studies of herbals extracts by classifying them into groups that show different degrees of cytotoxicity in relation to the content of their bioactive components.

## Figures and Tables

**Table 1:** Plant ingredients in the selected Thai traditional remedy and their extraction yield (% w/w of dried crude plant powder).

Botanical name	Family	Common name	Part used	% yield	Voucher number
*Acanthus ebracteatus* vahl.	Acanthaceae	Sea holly	Leaf	3.14	SKP 001 01 05 01
*Andrographis paniculata* (Burm.f.) Wall ex Nees.	Acanthaceae	Kariyat, Creat	Leaf	10.23	SKP 001 01 16 01
*Rhinacanthus nasutus* (L.) Kurz.	Acanthaceae	White crane flower	Leaf	17.90	SKP 001 18 14 01
*Ananas comosus* (L.)Merr.	Bromeliaceae	Pineapple	Fruit	3.65	SKP 029 01 03 01
*Commiphora myrrha* (NEES)Engler.	Burseraceae	Bullet Wood	Resin	15.01	SKP 031 03 13 01
*Carica papaya* L.	Caricaceae	Papaya	Root	9.64	SKP 040 03 16 01
*Artemisia vulgaris* Linn.	Compositae	Mugwort	Rhizome	3.29	SKP 051 01 22 01
*Mammea siamensis* Kosterm.	Calophyllaceae	Negkassar	Flower	5.62	SKP 216 13 19 01
*Mesua ferrea* Linn.	Calophyllaceae	Iron wood	Flower	4.71	SKP216 13 06 01
*Dioscorea opposite* thumb.	Dioscoreaceae	Nagaimo	Rhizome	3.14	SKP 062 04 15 01
*Equisetum arvense* L.	Equisetaceae	Horsetails	Leaf	1.45	SKP 224 05 01 01
*Croton stellatopilosus* Ohba.	Euphorbiaceae	Plow-noi (Thai)	Leaf	14.29	SKP 071 03 19 01
*Phyllanthus amarus* Schmach&Thonn.	Euphorbiaceae	Egg Woman.	Leaf	34.5	SKP 071 16 01 01
*Astragalus membranaceus* Bge. Var. monglolicu.	Fabaceae	Milkvetch Root	Root	9.12	SKP 072 01 13 01
*Heliotropium indicum* L.	Fabaceae	Alacransillo	Stem	2.98	SKP 072 08 09 01
*Enhalus acoroides* (L.f.) Royle.	Hydrocharitaceae	Sea Acorus	Root	2.18	SKP 088 05 01 01
*Ocimum tenuiflorum* L.	Lamiaceae	Holy Basil	Leaf	16.26	SKP 099 15 20 01
*Pogostemon cablin* (Blanco) Benth.	Lamiaceae	Patchoull	Stem	27.16	SKP 099 16 03 01
*Cinnamomum verum* J. Presl.	Lauraceae	Camphor	Bark	12.42	SKP 096 03 22 01
*Tamarindus indica* Linn.	Leguminosae	Tamarind	Leaf	12.5	SKP 098 20 09 01
*Ligusticum striatum* Dc.	Menispermaceae	Szechuan lovage	Rhizome	10.3	SKP 114 12 19 01
*Maclura cochinchinensis* Corner.	Moraceae	Cockspur thorn	Fruit	6.28	SKP 117 13 03 01
*Myristica fragrans* Houtt.	Myrisricaceae	Nutmeg tree.	Flower	12.57	SKP 121 13 06 01
*Nelumbo nucifera* Gaerth.	Nelumbonaceae	Sacred lotus	Root	1.89	SKP 125 14 14 01
*Jasminum sambac* Ait.	Oleaceae	Jasmine	Flower	20.1	SKP 129 10 19 01
*Flickingeria albopurpurea* Seidenf.	Orchidaceae	Fading Flickingeria	Flower	2.34	SKP 132 06 01 01
*Cardiospermum halicacalum* L.	Sapindaceae	Balloon vine	Vine	8.38	SKP 170 03 08 01
*Mimusops elengi* Linn.	Sapotaceae	Spash Cherry	Flower	6.92	SKP 171 13 05 01
*Schisandra chinensis.*	Schisandraceae	Schizandra Berry	Fruit	14.16	SKP 223 19 03 01
*Rehmannia glutinosa* (Gaertn.) libosch.	Scrophulariac	Chinese foxglove	Root	15.0	SKP 177 18 07 02
*Lycium barbarum.*	Solannaceae	Goji berry, Wolfberry	Leaf	9.81	SKP 180 12 02 01
*Solanum indicum* L.	Solannaceae	Black nightshade	Fruit	4.24	SKP 180 19 09 01
*Angelica sinensis* (Oliv.) Diels.	Umbelliferae	Dong quai	Bulb	2.5	SKP 199 01 19 01
*Foeniclum vulgare* mill subsp. piperatum (Ucr.) Beguinot.	Umbelliferae	Fennel	Fruit	3.07	SKP 199 06 22 01
*Linacia triadra* Miers.	Umbelliferae	Bai-ya-nang (Thai)	Rhizome	7.31	SKP 199 12 20 01
*Angelica dahurica* Benth.	Umbelliferae	African Tulip Tree	Bulb	6.23	SKP 206 01 04 01
*Vitis vinifera* L.	Vitaceae	Grape	Fruit	3.43	SKP 204 22 22 01
*Alpinia galangal* (L.) Willd.	Zingiberaceae	Glalanga.	Rhizome	1.78	SKP 206 01 07 01
*Amomum villosum* Lour var. Xanthiodes.	Zingiberaceae	Bastard cardamom,	Fruit	19.7	SKP 206 01 22 01
*Boesenbergia rotunda* (L.) Mansf.	Zingiberaceae	Fingerroot	Rhizome	9.62	SKP 206 02 18 01
*Curcuma aromatic* Salisb.	Zingiberaceae	Wild Turmeric	Rhizome	28.58	SKP 206 03 01 01
*Curcuma comosa* Roxb.	Zingiberaceae	Wanchakmodlook (Thai)	Bulb	12.96	SKP 206 03 03 01
*Kaempferia galanga* L.	Zingiberaceae	Aromatic ginger	Rhizome	1.14	SKP 206 11 07 01

**Table 2:** Cytotoxic activity (IC_50_; *μ*g/ml) of plant extracts against breast cancer (MCF-7), ovarian cancer (SKOV3), cervical cancer (HeLa), and human keratinocyte (HaCat) cell lines (mean ± SEM; *n* = 3).

Plant	Cytotoxicity (IC_50_ (*μ*g/ml) ± SEM)			
	MCF -7	SKOV3	Hela	HaCat
*A. ebracteatus*	>100	21.52 ± 1.28	25.31 ± 2.25	80.95 ± 0.94
*A. galanga*	34.79 ± 4.24	33.75 ± 1.21	34.88 ± 1.13	93.18 ± 2.20
*A. villosum*	30.66 ± 1.29	80.79 ± 3.82	59.42 ± 2.02	52.25 ± 0.58
*A. comosus*	>100	>100	>100	>100
*A. paniculata*	87.02 ± 3.21	>100	>100	38.29 ± 3.20
*A. sinensis*	34.15 ± 1.33	89.78 ± 1.25	72.25 ± 2.05	71.47 ± 2.55
*A. dahurica*	9.87 ± 2.13	>100	>100	>100
*A. valgaris*	49.16 ± 3.65	35.44 ± 1.07	46.54 ± 2.92	27.30 ± 3.71
*A. membranaceus*	>100	>100	>100	>100
*B. rotunda*	29.96 ± 1.09	35.05 ± 0.75	60.22 ± 2.15	34.25 ± 0.62
*C. halicacalum*	45.65 ± 2.32	>100	>100	>100
*C. papaya*	42.2 ± 2.11	>100	>100	>100
*C. verum*	20.88 ± 1.82	46.25 ± 1.25	24.12 ± 0.88	46.16 ± 1.43
*C. myrrha*	>100	>100	68.77 ± 4.10	>100
*C. sativus*	>100	>100	>100	>100
*C. stellatopilosus*	87.65 ± 3.21	>100	>100	34.21 ± 0.10
*C. aromatica*	90.43 ± 3.12	>100	>100	8.78 ± 0.21
*C. comosa*	68.56 ± 2.32	61.21 ± 1.26	73.21 ± 2.01	39.34 ± 0.96
*D. opposite*	>100	>100	>100	>100
*E. acoroides*	>100	>100	>100	>100
*F. albopurpurea*	14.63 ± 4.54	>100	>100	>100
*F. vulgare*	20.34 ± 2.64	45.27 ± 2.12	68.25 ± 1.85	>100
*H. indicum*	29.55 ± 3.21	35.13 ± 3.01	47.85 ± 2.02	>100
*E. arvense*	>100	>100	>100	>100
*J. sambac*	>100	>100	>100	>100
*K. galanga*	49.33 ± 1.45	>100	>100	>100
*L. triadra*	>100	46.25 ± 3.01	>100	81.53 ± 4.17
*L. striatum*	30.22 ± 2.32	28.32 ± 1.52	36.24 ± 2.20	>100
*L. barbarum*	>100	>100	>100	>100
*M. cochinchinensis*	53.47 ± 1.07	>100	>100	37.68 ± 0.85
*M. siamensis*	11.23 ± 2.82	>100	>100	33.39 ± 0.40
*M. ferrea*	86.42 ± 3.21	>100	>100	6.84 ± 0.39
*M. elengi*	>100	>100	>100	>100
*M. fragrans*	64.57 ± 2.22	>100	>100	32.58 ± 0.61
*N. nucifera*	>100	>100	>100	>100
*O. tenuiflorum*	50.56 ± 1.97	>100	>100	95.08 ± 0.59
*P. amarus*	23.23 ± 1.13	28.84 ± 2.15	35.21 ± 1.08	75.28 ± 1.40
*P. cablin*	24.65 ± 2.13	>100	>100	>100
*R. glutinosa*	>100	>100	>100	>100
*R. nasutus*	65.81 ± 3.28	48.28 ± 3.13	>100	>100
*S. chinensis.*	>100	>100	>100	>100
*S. indicum*	>100	>100	>100	>100
*T. indica*	14.76 ± 2.73	>100	>100	92.57 ± 0.94
*V. vinifera*	>100	>100	>100	>100
TTW	>100	>100	>100	47.12 ± 3.14
TTE	52.33 ± 2.05	>100	>100	>100

TTE = ethanolic extract of the Thai traditional cancer remedy; TTW = aqueous extract of the Thai traditional cancer remedy.

**Table 3:** Selectivity index (SI) of cytotoxic activity and total flavonoid content of the plant and remedy extracts.

Plant extracts	Selectivity index (SI)			Total flavonoid (mean ± SEM; mg quercetin eq./g)
	HaCat/MCF7 ratio	HaCat/SKOV3 ratio	HaCat/Hela ratio	
*A. ebracteatus*	0.81	3.76	3.20	151.32 ± 3.59
*A. galanga*	2.68	2.76	2.67	140.84 ± 4.47
*A. villosum*	1.70	0.65	0.88	79.72 ± 7.94
*A. comosus*	1.00	1.00	1.00	75.07 ± 4.14
*A. paniculata*	0.44	0.38	0.38	96.1 ± 5.87
*A. sinensis*	2.09	0.80	0.99	246.56 ± 2.58
*A. dahurica*	**10.13**	1.00	1.00	227.95 ± 5.69
*A. valgaris*	0.56	0.77	0.59	64.83 ± 3.47
*A. membranaceus*	1.00	1.00	1.00	185.91 ± 6.78
*B. rotunda*	1.14	0.98	0.57	175.38 ± 5.19
*C. halicacalum*	2.19	1.00	1.00	138.29 ± 3.98
*C. papaya*	2.37	1.00	1.00	134.27 ± 2.61
*C. verum*	2.21	1.00	1.91	220.97 ± 4.12
*C myrrha*	1.00	1.00	1.45	143.77 ± 4.37
*C. stellatopilosus*	0.39	0.34	0.34	46.38 ± 5.02
*C. aromatic*	0.10	0.09	0.09	259.7 ± 3.21
*C. Comosa*	0.57	0.64	0.54	139.28 ± 3.04
*D. opposite*	1.00	1.00	1.00	113.73 ± 5.71
*E. acoroides*	1.00	1.00	1.00	31.96 ± 2.29
*F. albopurpurea*	6.83	1.00	1.00	25.47 ± 2.13
*F. vulgare*	4.92	2.21	1.47	81.57 ± 2.97
*H. indicum*	3.39	2.85	2.09	33.64 ± 2.61
*E. arvense*	1.00	1.00	1.00	33.25 ± 2.42
*J. sambac*	1.00	1.00	1.00	30.06 ± 2.73
*K. galanga*	2.03	1.00	1.00	88.23 ± 2.71
*L. triadra*	0.82	1.76	0.82	136.84 ± 2.68
*L. striatum*	3.31	3.53	2.76	34.39 ± 3.07
*L. barbarum.*	1.00	1.00	1.00	85.6 ± 2.98
*M. cochinchinensis*	0.71	0.38	0.38	93.77 ± 3.4
*M. siamensis*	2.97	0.33	0.33	204.94 ± 2.72
*M. ferrea*	0.08	0.07	0.07	216.49 ± 7.31
*M. elengi*	1.00	1.00	1.00	190.5 ± 3.54
*M. fragrans*	0.51	0.33	0.33	72.23 ± 3.59
*N. nucifera*	1.00	1.00	1.00	82.43 ± 2.53
*O. tenuiflorum*	1.88	0.95	0.95	124.35 ± 2.74
*P. amarus*	3.24	2.61	2.14	164.6 ± 6.21
*P. cablin*	4.06	1.00	1.00	73.58 ± 4.98
*R. glutinosa*	1.00	1.00	1.00	101.58 ± 2.06
*R. nasutus*	1.52	2.07	1.00	183.57 ± 5.46
*S. chinensis.*	1.00	1.00	1.00	94.79 ± 5.57
*S. indicum*	1.00	1.00	1.00	163.17 ± 3.44
*T. indica*	6.27	0.93	0.93	158 ± 5.50
*V. vinifera*	1.00	1.00	1.00	14.8 ± 2.73
TTW	0.47	0.47	0.47	41.92 ± 4.04
TTE	1.91	1.00	1.00	105.67 ± 4.33

## Data Availability

The data supporting the conclusions of the study are available from the corresponding author upon request.
